# Dental Materia Medica

**Published:** 1866-01

**Authors:** Geo. Watt


DENTAL REGISTER.

VOL. XX.] JANUARY, 1866. [No. 1.

### Original Communications.

### DENTAL MATERIA MEDICA.

### NITRATE OF SILVER, AgO, $NO_5$

BY GEO. WATT.

THIS salt is usually prepared from silver coin, which is an alloy of silver and copper. After the coin is dissolved in nitric acid, the copper is removed by a special chemical process. But when a dentist prepares it, his best course is to purify the silver before dissolving it in the acid.

PREPARATION.—Dilute one ounce of nitric acid with two ounces of pure water, and add, in thin slips, as much silver as it will dissolve. Evaporate the solution to dryness, and the result is crystallized nitrate of silver. If desired, the crystals may be melted, slowly, in a crucible or porcelain capsule, and poured into iron molds.

But dentists will have but little occasion to prepare the nitrate, as it can be obtained in any drugstore, or from any regular physician.

PURITY.—Prepared as above, the salt is pure; but much of it, as found in market, is otherwise. The salt is white, and completely soluble in distilled water. It blackens, from partial reduction, in contact with organic matter. When it is contaminated with nitrate of copper, the addition of concentrated ammonia to its solution develops a blue color. By adding hydrochloric acid, or a solution of common salt to its solution, a white precipitate falls, which dissolves readily in caustic ammonia when the salt is pure, but not when lead is present. When the white precipitate (chloride of silver) is all thrown down, the watery solution remaining should evaporate without a residuum. If any is left, it is due to adulteration.

PROPERTIES.—Nitrate of silver forms colorless, transparent, prismatic crystals. It has a bitter, metallic taste. It fuses at a temperature of 425°, and decomposes at 600° or 700°. It does not deliquesce; nor does it change color in the light, except when organic matter is present. In contact with organic matter, a part of it is decomposed, and suboxyd of silver is deposited. It is soluble in water and in alcohol. Its affinity for organic matter is so strong that it is a very reliable test for its presence, and the readiness with which its black suboxyd is perceived, renders it all the more convenient for this purpose.

PHYSIOLOGICAL EFFECTS.—Nitrate of silver is a most powerful corrosive poison. Its local action is that of a caustic, or escharotic. Dr. Turner imputes this power to the action of the nitric acid, which is liberated by the decomposition of the salt, from its contact with organic matter. But this explains only a part of the process; for the salt has a strong affinity for albumen, fibrin and casein. It unites with the former of these, according to Lassaigne, in the proportion of 84.5 of albumen to 15.5 of the nitrate. Its union with the others is in the same proportions. The compound of the nitrate with albumen is soluble in an excess of either ingredient, and also in ammonia and chloride of sodium.

Nitrate of silver, in common with other escharotics, produces a change in the vital actions of the surrounding tissues, resulting in exalted vitality, and often in inflammation. Its action on living matter is, therefore, *chemico-vital.*

When the nitrate is applied to the skin, or an ulcerated surface, a whitish compound results, formed by a union of the salt with the albumen of the tissue. This soon turns black, owing to the decomposition of the nitrate, and the reduction of the protoxyd of silver to a suboxyd. Though on account of its very strong affinity for albuminous tissues, the nitrate is a powerful caustic; yet, ordinarily, the pain produced by its action is not very severe. This results from two causes: By the energy of its combination with the albumen of the tissue to which it is applied, the vitality of the surface is almost instantly destroyed; and the compound of the salt and the albumen, being insoluble, in most substances, acts as an elastic and perfectly fitting covering to the subjacent living parts, protecting them from the action of the atmosphere and other irritants. This devitalized layer hardens, corrugates, and in a few days, peels off; usually leaving the parts beneath in a healthier state than before the application.

Uses.—From what has been said of the chemical and physiological effects of the nitrate, the intelligent practitioner will be at no loss in regard to its practical application. It is used by dentists to relieve sensitive dentine, as an application to diseased gums, and to arrest hemorrhage.

Hypertrophied gums are benefited by the nitrate applied in substance; and the same is true of tumors and callous growths on the gums. Apthous ulcers are often promptly relieved by the nitrate; but there are other remedies better adapted to their treatment.

When the nitrate is applied to dentine it acts promptly, by uniting with the gelatinous portion, destroying its vitality to the extent of the combination. A part of the salt is decomposed; and the liberated acid combines with the calcareous materials. The suboxyd of silver, and the compound of the nitrate with albumen, both being insoluble, there can be no danger of the salt, or its results, being absorbed by the dentine. The fear of discoloring the tooth, by this means, as expressed by Prof. McQuillen and others, is groundless.

The nitrate acts on dentine with more energy than chloride of zinc, but with less pain, and not to so great a depth.

Nitrate of silver is used extensively, both by dentists and physicians, to arrest hemorrhage; but in this respect, its use is almost empirical. It is not entitled to rank as a styptic. It forms a coagulum with the albumen and fibrin of the blood; but this clot is, as already remarked, soluble in albumen; hence the blood dissolves its way through it, and the hemorrhage is resumed. It is true that this agent often succeeds; but others are far more reliable. A solution of the nitrate is sometimes used as a wash for the mouth, in ptyalism, or when there is a spongy condition of the gums; but it has no advantages over astringents or detergents, and is objectionable on account of the suboxyd depositing on and blackening the teeth. The dentist has no occasion to prescribe its internal use.

### CREOSOTE, $C_{14} H_9 O_8$.

Creosote was discovered by Reichenbach, who gave it its present name on account of its remarkable antiseptic power. The word means *the preserver of flesh.*

Creosote is obtained by the destructive distillation of wood, or other organic matter. Its preparation is complex, and need not be detailed here. The curious reader is referred to the Dispensatory, Turner's Chemistry, or other standard works.

PROPERTIES.—Pure creosote is an oleaginous, colorless, transparent liquid, with a smoke like odor, and a caustic, burning taste, "with a sweetish after taste." Its specific gravity is 1.03, according to its discoverer, and 1.06 according to Dr. Christison. This discrepancy doubtless results from the impurity of one or both of the specimens experimented on. Indeed, the composition of creosote is rather uncertain, but is probably about as indicated by the above symbols, Its boiling point is 397°. It forms a resinified compound with chlorine, decomposes nitric acid, and is decomposed by it, and is soluble in alcohol, ether, acetic acid, naptha, and sulphuret of carbon. It dissolves resins, and some of the metalloids, as an example, iodine. It forms two definite combinations with water. One of these contains 100 parts of creosote and 10 of water. The other, 1.25 of creosote and 100 of water. It forms a coagulum with albumen, but appears to have but little affinity for fibrin. Its affinity for albumen and its ability to form an insoluble compound with it constitute the secret of its antiseptic power.

PURITY.—Pure creosote is colorless, and does not become colored by exposure to light. It is completely soluble in acetic acid and in a solution of caustic potash. If not dissolved by these, it is adulterated.

PHYSIOLOGICAL EFFECTS.—Creosote is an active caustic. Its escharotic power, as stated above, is due to its affinity for albumen, which is so strong as to take the latter from living tissue, and thus destroy vitality to the extent of the combination. Its compound with albumen is white, hence the extent of its escharotic action is readily observed.

USES.—Creosote is much more useful to the dentist than to the physician. For the relief of toothache resulting from exposure of the pulp, it is the sovereign remedy. By devitalizing the surface of the pulp, and forming an elastic, flexible, and insoluble layer over the subjacent living parts, it usually gives complete, and almost instantaneous relief. By applying it around the neck of a tooth, by means of a soft string, or otherwise, the pain of dental periostitis is often allayed. In the cure of alveolar abscess, it is invaluable. The sac at the end of the dental root may be totally obliterated by passing creosote into it, either through the canal or by an opening made through the gum and process. Its combination with iodine is valuable in this respect, especially with scrofulous and syphilitic patients. Properly diluted, it is a good application, when supuration has taken place within the antrum. When the dentist desires an irritant, an escharotic, or an antiseptic, creosote will not disappoint him. All its value depends on its ability to perform these important offices.

A strange fallacy in regard to creosote has almost universal credence. I allude to the belief that it promotes the decay of the teeth, Many physicians, (I fear, a majority), are blinded by this delusion, than which nothing can be further from the truth. They believe that creosote causes decay of the teeth; yet they smoke their bacon, *to impregnate it with creosote*, to prevent its decay.

Creosote is a better styptic than nitrate of silver, but is less reliable than tannin, or the perchloride, or persulphate of iron.

I know of nothing, in the province of dentistry, that calls for the internal use of creosote.

